# Comparison of Selection Traits for Effective Popcorn (*Zea mays* L. var. Everta) Breeding Under Water Limiting Conditions

**DOI:** 10.3389/fpls.2020.01289

**Published:** 2020-08-27

**Authors:** Samuel Henrique Kamphorst, Antônio Teixeira do Amaral Júnior, Valter Jário de Lima, Pedro Henrique Araújo Diniz Santos, Weverton Pereira Rodrigues, Janieli Maganha Silva Vivas, Gabriel Moreno Bernardo Gonçalves, Katia Fabiane Medeiros Schmitt, Jhean Torres Leite, Marcelo Vivas, Freddy Mora-Poblete, Omar Vergara-Díaz, Jose Luis Araus Ortega, José Cochicho Ramalho, Eliemar Campostrini

**Affiliations:** ^1^ Laboratório de Melhoramento Genético Vegetal, Centro de Ciências e Tecnologias Agropecuárias, Universidade Estadual Norte Fluminense Darcy Ribeiro (UENF), Rio de Janeiro, Brazil; ^2^ Centro de Ciências Agrárias, Naturais e Letras, Universidade Estadual da Região Tocantina do Maranhão, Estreito, Brazil; ^3^ Instituto de Ciencias Biológicas, Universidad de Talca, Talca, Chile; ^4^ Unitat de Fisiologia Vegetal, Facultat de Biologia, Universitat de Barcelona, Barcelona, Spain; ^5^ AGROTECNIO (Center of Research in Agrotechnology), Lleida, Spain; ^6^ PlantStress & Biodiversity Lab, Centro de Estudos Florestais (CEF), Departamento de Recursos Naturais, Ambiente e Território, Instituto Superior de Agronomia, Universidade de Lisboa, Oeiras, Portugal; ^7^ Unidade de Geobiociências, Geoengenharias e Geotecnologias (GeoBioTec), Faculdade de Ciências e Tecnologia (FCT), Universidade NOVA de Lisboa (UNL), Monte de Caparica, Portugal

**Keywords:** carbon isotope composition (δ^13^C), drought, leaf gas exchanges, popcorn yield, water use efficiency

## Abstract

Climate change is expected to intensify water restriction to crops, impacting on the yield potential of crops such as popcorn. This work aimed to evaluate the performance of 10 field cultivated popcorn inbred lines during two growing seasons, under well-watered (WW) and water stressed (WS) (ψ_soil_≥ −1.5 MPa) conditions. Water stress was applied by withholding irrigation in the phenological phase of male pre-anthesis. Additionally, two contrasting inbred lines, P7 (superior line) and L75 (low performer) were compared for grain yield (GY) and expanded popcorn volume (EPV), selected from previous studies, were tested under greenhouse conditions. In the field, no genotype x water condition x crop season (G×WC×CS) interaction was observed, whereas GY (−51%), EPV (−55%) and leaf greenness (SPAD index) measured 17 days after anthesis (DAA) (> −10%) were highly affected by water limitation. In general, root traits (angles, number, and density) presented G×WC×CS interaction, which did not support their use as selection parameters. In relation to leaf senescence, for both WS and WW conditions, the superior inbred lines maintained a stay-green condition (higher SPAD index) until physiological maturity, but maximum SPAD index values were observed later in WW (48.7 by 14 DAA) than in WS (43.9 by 7 DAA). Under both water conditions, negative associations were observed between SPAD index values 15 and 8 days before anthesis DBA), and GY and EPV (r ≥ −0.69), as well as between SPAD index 7, 17, and 22 DAA, and angles of brace root (AB), number of crown roots (NC) and crown root density (CD), in WS (r ≥ −0.69), and AB and CD, in WW (r ≥ −0.70). Lower NC and CD values may allow further root deepening in WS conditions. Under WS P7 maintained higher net photosynthesis values, stomatal conductance, and transpiration, than L75. Additionally, L75 exhibited a lower (i.e., more negative) carbon isotope composition value than P7 under WS, confirming a lower stomatal aperture in L75. In summary, besides leaf greenness, traits related to leaf photosynthetic status, and stomatal conductance were shown to be good indicators of the agronomic performance of popcorn under water constraint.

## Introduction

Since the pre-industrial period, anthropogenic activities mainly associated with increased greenhouse gas emissions, have caused an increase of about 1°C in the average global temperature. Depending on future emissions, this increase may reach up to 1.5°C between 2030 and 2052, which tends to aggravate the frequency of extreme droughts and rainfall events worldwide (IPCC, 2019). This will have severe impacts on world agribusiness (Awange et al., 2016; Van Loon et al., 2016), particularly for Brazil, whose economy relies on large-scale agricultural activities. Thus, global environmental changes may increase plant stress levels, requiring specific plant breeding expertise to overcome these challenging conditions (Blum, 2011).

Popcorn sales are worth approximately one hundred million dollars annually in the USA, and there is also a high demand in Brazil for this product (Kist et al., 2019). The seed market offers cultivars with high-yield potential for environments with optimal water supply but, to date, there are no cultivars adapted to water deficit conditions, with droughts being one of the most limiting abiotic factors for the productivity of this crop (Kamphorst et al., 2019; Lima et al., 2019). Thus, it is essential to obtain genotypes which are more efficient regarding water use and identifying plant traits for genotypic selection under drought conditions.

The initial step is the evaluation of germplasm with identification of predictor characteristics for drought adaptation. Traditionally, secondary characteristics associated with higher grain yield are considered, such as a shorter interval between male and female anthesis (Câmara et al., 2007; Teixeira et al., 2010), late leaf senescence (Câmara et al., 2007; Costa et al., 2008), higher prolificity (Li et al., 2003; Câmara et al., 2007), and fewer tassel branches (Durães et al., 2004; Câmara et al., 2007). Technological advances associated with the use of remote sensing techniques present the opportunity for using new approaches such as canopy temperature measurements (Romano et al., 2011; Zia et al., 2013), leaf senescence estimated by NDVI (Cabrera-Bosquet et al., 2011; Adebayo et al., 2014), leaf greenness, obtained by SPAD index (Cairns et al., 2012), and RGB images (Araus et al., 2018).

Photosynthesis is the process responsible for increasing plant biomass and productivity, while stomatal conductance is responsible for controlling water loss to the atmosphere, however reducing stomatal opening also decreases CO_2_ availability to the RuBisCO carboxylation sites, and thus C-assimilation (Farquhar and Sharkey, 1982; Buckley, 2019). Therefore, gas exchange measurements can be used to distinguishing tolerant/susceptible genotypes to drought (Flexas et al., 2018), however this requires a high number of evaluations under field conditions in breeding programs (Earl and Tollenaar, 1999). Additionally, the characteristics derived from leaf spectral indices associated with canopy temperature, cell and leaf water content, and the composition of photosynthetic leaf pigments, allows the use of thermal and multispectral imaging as feasible options to understand biological phenomena and select plants under stress (Fernández-Calleja et al., 2020). Another option is the isotopic signature obtained from C^13^ quantification, which indicates water use efficiency (Araus et al., 2010; Chairi et al., 2016). The deployment of these high-performance phenotyping techniques is known as physiological breeding (Reynolds and Langridge, 2016).

Some of the high-performance phenotyping techniques use multispectral analyses associated with photosynthetic leaf pigment (NDVI, PRI) and chlorophyll concentrations (SPAD index) (Araus et al., 2018). The analysis of these characteristics has been efficiently used to select drought tolerant genotypes (Khanna et al., 2019), associated with the fact that genotypes which are more susceptible to soil water limitation, present increased leaf senescence (Romano et al., 2011; Araus et al., 2012; Zia et al., 2013). Both leaf and canopy studies of leaf senescence can generate information to improve the ability to estimate grain yield (Echarte et al., 2008; Escobar-Gutiérrez and Combe, 2012). Stay-green maize genotypes are the most productive and considered more adapted to drought conditions. However, the efficient use of phenotyping methodologies partly depend on the ability to use them accurately at critical grain production stages (Cairns et al., 2012; Adebayo et al., 2014; Araus et al., 2018).

Maize plants under water stress have less crown roots, increasing water acquisition through other roots located in deeper soil layers (Gao and Lynch, 2016), since a deeper root system can allow access to water in additional soil layers (Hund et al., 2009). Therefore, in cases of soil water restriction, an efficient root architecture associated with a deeper root system, is essential to delay leaf senescence, maintaining a high photosynthetic performance to sustain growth and productivity (Lynch, 1995). In this context, the objective of this study was to evaluate the field performance of agronomic and root characteristics and the SPAD index of a group of popcorn inbred lines cultivated under contrasting soil water conditions during male pre-anthesis: well-watered (WW) and water-stressed (WS). Furthermore, this study evaluated the use of possible players (selection traits) in the selection of contrasting grain yield (GY) and expanded popcorn volume (EPV) genotypes cultivated under greenhouse conditions.

## Materials and Methods

### Field Experiment Conditions

#### Plant Material

This study evaluated ten popcorn inbred lines (S_7_), whose genealogy is derived from germplasm adapted to tropical (L61, L63, L65, and L71 from the BRS-Angela population) and temperate/tropical conditions (P7, from the commercial hybrid Zélia; P2 and P3, from the compound CMS-42; P6, from the commercial hybrid IAC-112; L54, L55, and L75 and L76, from the Barão de Viçosa population) (Kamphorst et al., 2019).

#### Experimental Design, Cultural Treatments, and Water Conditions

The experiments were conducted at the Experimental Station of the Antônio Sarlo Agricultural State College in Campos dos Goytacazes, RJ, Brazil (Latitude 21°42′48″ S, Longitude 41°20′38″ W; altitude 14 m), in the crop seasons (CS) of 2016 and 2018, during the dry seasons (April and August).

The experimental design was completely randomized blocks with three repetitions for both well-watered (WW) and water-stressed (WS) conditions. The plots comprised two 4.40-m lines, spaced 0.20 m between plants and 0.80 m between lines (44 plants per plot).

**Figure 1:**
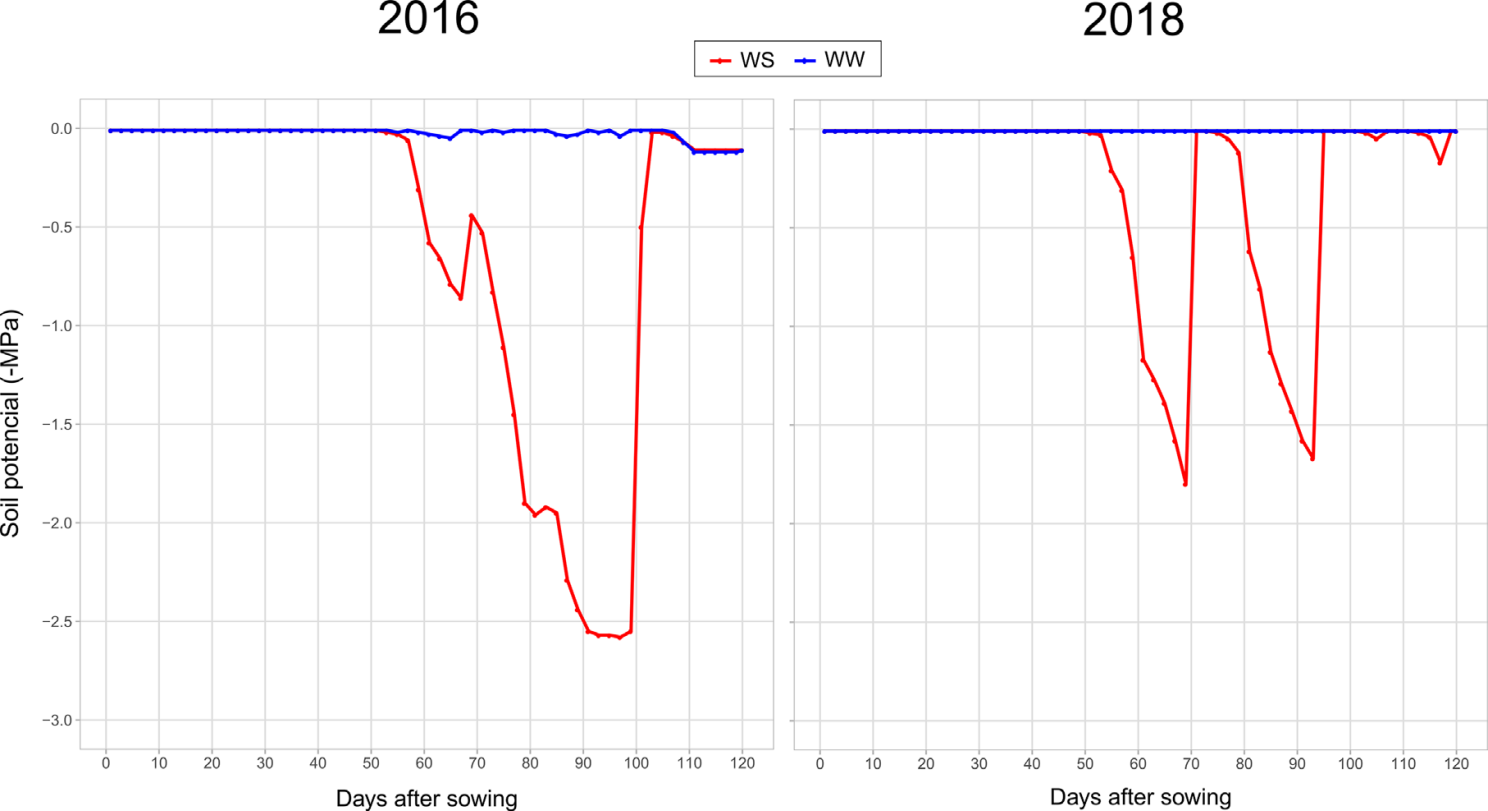
Soil water potential (MPa) in days after sowing for experiments performed during the 2016 and 2018 harvests with popcorn inbred lines in water stressed (WS, in red) and well-watered (WW, in blue) conditions.

The plants were irrigated using a drip system, using a Katif dripper for each plant at a flow rate of 2.3 mm h^−1^. Soil water was monitored using three Decagon MPS-6 sensors (Decagon, USA) installed at a depth of 0.20 m in the planting line between two plants. The WW condition received irrigation at soil field capacity (−0.01 MPa), whereas in WS conditions, irrigation was withheld 15 days before male anthesis (June 22nd to June 26th during both years). Under WS, the soil reached permanent wilting point (−1.5 MPa) 77 days after sowing (DAS) (grain filling stage) in the 2016 experiment, and at 68 DAS (male anthesis) and 90 DAS (grain filling stage) in the 2018 experiment (due to a rainfall episode between these two periods) (**Supplementary Table 1**). During the experimental period, total rainfall was 133 and 148 mm in 2016 and 2018, respectively (**Supplementary Table 1**, **Figure 1**).

**Figure 2:**
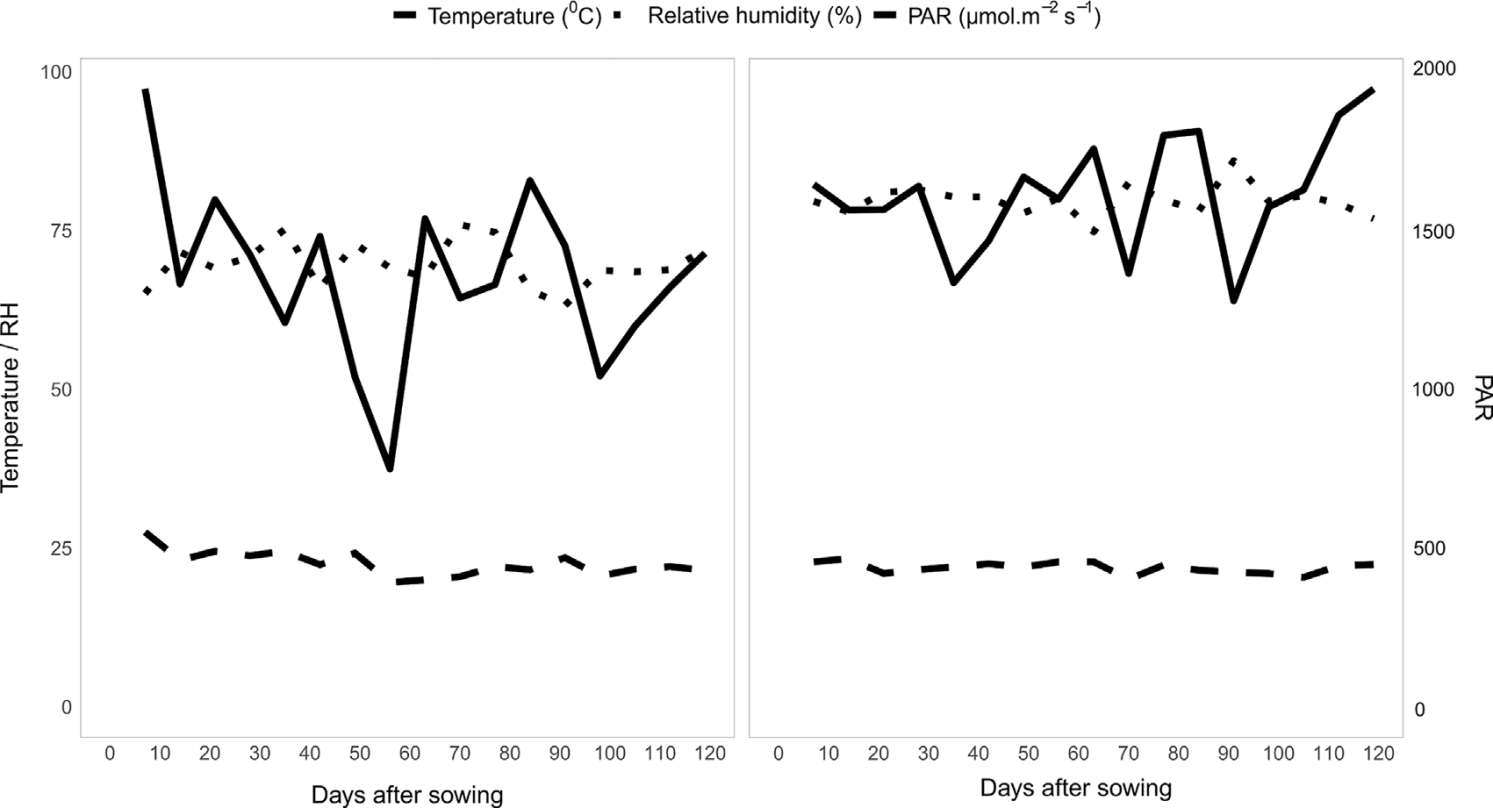
Weekly average values on DAS of temperature (°C), relative humidity (%) and solar radiation (µmol m^−2^s^−1^) throughout the growing period of the experiments with popcorn inbred lines during the 2016 and 2018 crop seasons.

During the crop cycle, in the 2016 and 2018 harvests, the average temperature was 22.6°C and 21.6°C and the relative humidity was 69.7% and 78.5%, respectively. The average solar radiation was ≅1,100 and 1,200 µmol m^−2^ s^−1^ in 2016 and 2018, respectively (**Figure 2**). Weather conditions were recorded at a National Institute of Meteorology (INMET) weather station located near the experimental area.

Planting fertilization included 30 kg ha^−1^ N (urea), 60 kg ha^−1^ P_2_O_5_ (triple superphosphate), and 60 kg ha^−1^ K_2_O (potassium chloride). Cover fertilization (30 DAS) included 100 kg ha^−1^ N (urea).

#### Agronomic and Root System Traits and the SPAD Index

The 100-grain weight (100GW) was determined by averaging the weight (g) of two samples of 100 grains per plot. Popping expansion (PE) was measured by the mass of 30 g of grains placed in a microwave oven in a kraft bag for 2 min, with the volume of popcorn quantified in a 2000-ml beaker and the ratio of the popped volume being divided by 30 g and expressed in ml g^−1^. Grain yield (GY) was obtained after threshing the ears of each plot, which were corrected to 13% humidity (kg ha^−1^). The EPV was obtained by multiplying GY and PE (m^3^ ha^−1^). The row number per ear (RNE) and grain number per row (GNR) were determined by counting, ear diameter (ED) was estimated with a Vernier caliper (mm), and ear length (EL) was measured with a ruler (cm). The characteristics 100GW, PE, GY, and EPV were measured in all plants in the plot. GNR, RNE, ED, and EL were measured using a random sample of six plants per plot.

Root architecture was evaluated based on the methods used by Trachsel et al. (2011) with some modifications. The root systems of two plants per plot, one from each row, were removed in a 40 cm diameter soil cylinder with a depth of 25 cm to allow the following measurements: angles (°) of brace (AB) and crown roots (AC) [using a protractor and expressed in relation to the soil]; number (N) of adventitious (NA), of brace (NB), and of crown roots (NC) [by counting these structures]; and branching density (D) of adventitious (DA), of brace (DB), and of crown roots (DC) [using the diagrammatic scale proposed by Trachsel et al. (2011)]. Root density values ranging from 1 to 9, with higher counts indicating higher density.

Leaf greenness (SPAD index) was estimated using three readings from the middle third of the leaf counted from the apex and below the flag leaf using a portable SPAD-502 chlorophyll meter (Minolta, Japan). The SPAD index was measured on eight different dates according to male anthesis: 15 (S1) and 8 (S2) days before anthesis (DBA), during male anthesis (S3), and 7 (S4), 17 (S5), 22 (S6), 35 (S7), and 42 (S8) days after anthesis (DAA). SPAD index was evaluated in six plants per plot, with three readings per plant.

#### Statistical Analysis

Analysis of variance was performed for each water treatment and year considering the linear model: y_ij_ = µ + g_i_ + b_j_ + e_ij_, where mean (µ) and genotype effect (g) were considered fixed and block (b) and error (e) were considered random.

The experiments were then jointly analyzed considering different water treatments and years using the following mathematical model: y_ijk_ = µ + (b/l)/wc_jkm_ + g_i_ + cs_j_ + wc_k_ + g_aij_ + gw_cik_ + csw_cjk_ + gcswc_ijk_ + e_ijk_, where the mean (µ), the effect of genotype (G), crop (CS), water condition (WC), G × CS interaction (gcs), G × WC interaction (gwc), CS × WC (cswc) interaction, and G × CS × WC interaction (gcswc) were considered fixed and the block within WC (b/l) and error (e) were considered random.

The variables that showed significant differences between genotypes with at least 5% probability from the F-test were analyzed with the Tukey’s test at the same level of significance.

Regression analysis was performed for the SPAD index variable after analysis of variance using the quadratic model and considering significance at 5% probability from the F-test.

Subsequently, genotypic correlation coefficient (*rg*) estimates were calculated according to Mode and Robinson (1959), and tested at 5% and 1% probability levels using the *t*-test.

### Greenhouse Experiments

#### Genotypes and Growth Conditions

This experiment was conducted from early May to mid-July 2019 (spring-summer), in a greenhouse at the Faculty of Biology, University of Barcelona, Spain, using two inbred lines (P7 and L75) identified as drought tolerant and sensitive, respectively, in the 2016 and 2018 field experiments in Brazil. Three seeds from each lineage were germinated in substrate in PVC tubes of 14 cm diameter and 150 cm length, which were kept at field capacity (FC). The substrate consisted of 80% perlite and 20% peat with the addition of a slow-release fertilizer, which in absolute values corresponded to 144.7 kg N ha^−1^, 36.2 kg P ha^−1^, and 57.8 kg K ha^−1^.

The tubes were longitudinally divided into two halves held together with adhesive tape. The lower part of the tube was tied with wire and closed with a vase of the same diameter, with eight holes to avoid loss of substrate. In addition, the tubes were covered with plastic to reduce evapotranspiration.

**Figure 3:**
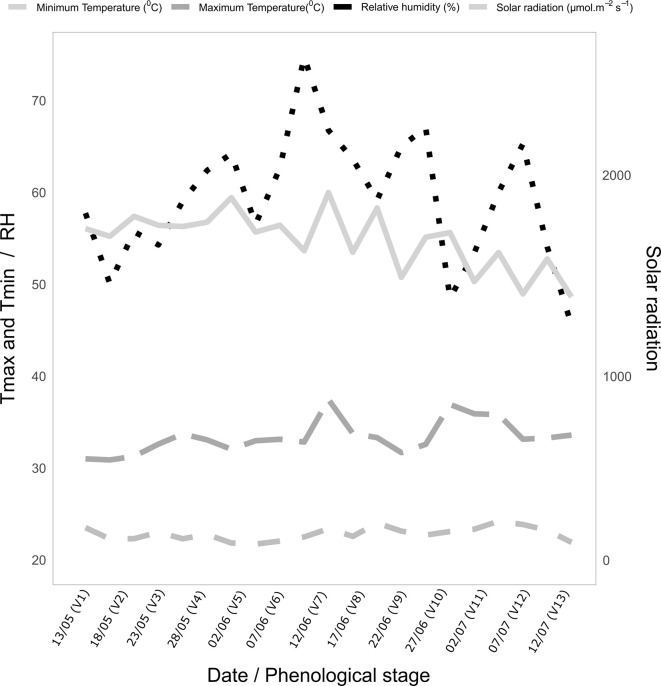
Average minimum (Tmin) and maximum (Tmax) air temperatures (°C), relative humidity (RH, %), and photosynthetically active radiation (PAR, µmol m² s¹) as a function of the phenological stages (V) during the growing period of popcorn plants.

Thinning was performed 15 days after germination, retaining one plant per tube. The experiment was arranged in completely randomized blocks under the two water conditions, with three repetitions each. The plants were cultivated under a rain shelter that allowed the control of water availability. The temperature and humidity inside the greenhouse followed a seasonal pattern as shown in **Figure 3**.

For field capacity evaluation the tubes were fully irrigated before plant sowing, and left for 72 h to drain the excess irrigation water. After this time, the cylinders were weighed. Field capacity was calculated from the weight difference between the dry substrate and after draining excess water.

In the WW condition, the tubes were kept at FC until the time of the evaluations, which were conducted during the pre-anthesis period. For this, every 2 to 3 days before irrigation, the tubes were weighed and received the corresponding amount of water to return to FC. In the WS condition, limited irrigation was imposed 15 days after the emergence of seedlings. The amount of water available in the tubes gradually reduced according to plant consumption, until reaching 35% of FC (40 DAS); thereafter, they were maintained for 15 days until harvest. The decrease in moisture in the tubes was homogeneous as the plants that consumed the most water received the precise amount of irrigation to return to the substrate water conditions of the plants that consumed the least. In both WCs, the weight of the plants was disregarded for the calculation of the water capacity of the tubes.

#### Morphological Characteristics, Stomata and Epidermal Cell Density, and Foliar Pigments

After harvest, plants were collected and dried (70°C, 72 h) to determine shoot biomass (SB; g). The specific leaf area (SLA) was calculated using the leaf area (cm²) to dry leaf mass (cm ^2^ g^−1^) ratio. For this, of 1.65 cm diameter leaf discs were collected from the last developed leaf of each plant and dry mass determined as above.

The leaf area (m²) was calculated from photographs of each individual plant. The images were acquired with a Sony α6000 DSLR (Sony Corporation, Japan), 24.5-megapixel resolution camera with a 23.5 x 15.6 mm sensor size, native resolution of 6,000 × 4,000 pixels and equipped with a 35-mm focal length lens. The pixel size was calculated using the Ground Sample Distance (GSD) calculator tool developed by Pix4D (https://support.pix4d.com/hc/en-us/articles/202559809-Ground-sampling-distance-GSD). We have used the following equation: $D=\frac{\left(Sw.H.100\right)}{\left(Fr.imW\right)},$ where GSD represent the distance between two consecutive pixel centers, Sw denotes the sensor width of the camera (mm), H is the height distance between the camera and the object (m), Fr is the real focal length of the camera (mm) and imW is the image width (pixels). In our case, Sw = 23.2 mm, the height average was H = 1.95 m, Fr = 18 mm and the imW = 4608 pixels. Therefore, images with GSD = 0.054 cm/pixel were analyzed using ImageJ software (Schneider et al., 2012).

For the evaluation of stomatal morphological traits, the adaxial and abaxial epidermal sides of the last developed leaf (2nd or 3rd leaf counted from the apex specifically between the central vein and the extremity), were coated with a colorless nail base coat. After drying for 10 min, the dried coat layer was removed with adhesive tape and transferred to a glass slide. The number of stomata (s) and epidermal cells (e) were counted at ×40 magnification. Each foliar replica (adaxial and abaxial) was counted in three microscope fields, according to the methodology described by Radoglou and Jarvis (1990).

The stomatal and epidermal cell densities were calculated using the following equations: $\text{SD}=\frac{\text{s}}{0.152}$ and $\text{ECD}=\frac{\text{e}}{0.152},$ where SD is stoma density (mm^−2^), ECD is epidermal cell density (mm^−2^), and 0.152 mm^−2^ is the surface area of the microscope (0.22 mm radius).

The stomatal index (SI, %) of each leaf face was calculated using the following equation: $\text{SI}=100\cdot \left(\frac{\text{SD}}{\text{ECD}}\right).$

Foliar chlorophyll, flavonoid, and anthocyanin epidermal levels and the nitrogen balance index were evaluated from the middle third of the last leaf developed at the time of harvest, using a portable Dualex^®^ meter (FORCE-A, Orsay, France).

#### Foliar Gas Exchange and Fluorescence Measurements, Relative Foliar Water Content, and C Isotope Discrimination

One day before harvest, between 11:00 am and 2:00 pm, gas exchanges were evaluated using an infrared gas analyzer, model LI-6400 (LI-COR, Lincoln, NE, USA), equipped with a light source (6400-40 LCF, LI-COR). During the evaluations, the PAR was set to 1500 μmol m^−2^ s^−1^, CO_2_ concentration to 400 µmol mol^−1^, relative humidity to between 55% and 60%, and temperature to 25°C. Net photosynthetic rate (A), stomatal conductance (g_s_), and transpiration rate (E) were evaluated from the last developed leaf of each plant. The instantaneous (WUE_instant_ = A/E) and intrinsic (WUE_intrinsic_ = A/g_s_) water use efficiencies were also calculated.

The agronomic water use efficiency was calculated using the equation ${\text{WUE}}_{\text{agron}}=\frac{\text{SB}}{{\text{T}}_{\text{cum}}}$, where SB is the shoot dry biomass, and T_cum_ is the total amount of transpired water of each plant (T_cum_, dm^3^ plant^−1^). Irrigation was controlled during the whole growth cycle, which allowed estimation of the T_cum_ values.

The stable carbon (^13^C:^12^C) isotope ratio was measured in leaf dry matter using an elemental analyzer (Flash 1112 EA; Thermo Finnigan, Bremen, Germany) coupled to an isotope ratio mass spectrometer (Delta C IRMS, Thermo Finnigan) operating in a continuous flow mode. Samples of 0.7–0.8 mg of leaf dry matter from each plant, together with reference materials were weighed and sealed into tin capsules. Measurements were performed at the Scientific Facilities of the University of Barcelona. Isotopic values were expressed in composition notation (δ) as follows: δ^13^C = [(^13^C/^12^C) sample/(^13^C/^12^C) standard]–1, where “sample” refers to plant material and “standard” to international secondary standards of known ^13^C:^12^C ratios (IAEA CH7 polyethylene foil, IAEA CH6 sucrose, and USGS 40 L-glutamic acid) calibrated against Vienna Pee Dee Belemnite calcium carbonate with analytical precision (standard deviation) of 0.15‰. The last fully developed leaf of each plant was used.

Before harvesting, leaf discs (1.65 cm in diameter) were collected from the same leaves as those used for gas exchange evaluation to obtain the relative water content according to the methodology proposed by González and González-Vilar (2001).

#### Root Traits

After harvesting the shoots, the roots were also collected, being abundantly washed with water to remove perlite and peat, rinsed with distilled water and superficially dried with paper towels. Finally, the root material was cut into five equal sections, each 0.30 m long, obtained from the upper surface of the tubes to the lower end, in the following layers: 0–30 cm (a), 30–60 cm (b), 60–90 cm (c), 90–120 cm (d), and 120–150 cm (e).

The root sections were stored separately in a paper envelope and dried (70°C, 72 h). Afterward, the ratio between the dry biomass of the aerial parts and the roots was calculated.

The root weight density in each section of soil (RWD_sec_, g m^−3^) was estimated by: ${\text{RWD}}_{\text{sec}}={\text{RB}}_{\text{sec}}/(\pi *{\text{R}}^{2}*\text{L})$ where RB_sec_ is the dry biomass of the roots in the soil section (g); R is the tube radius (0.07 m), and L is the length of the section (0.30 m long) (Elazab et al., 2012).

#### Statistical Analysis

Analysis of variance was performed for each water condition (WS and WW) and for the interaction genotype by WC. The analysis followed the statistical model: ${\text{Y}}_{\text{ijk}}=\text{μ}+{\text{G}}_{\text{i}}+\text{B}/{\text{WC}}_{\text{jk}}+\text{WC}+{\text{WC}}_{\text{ij}}+{\text{ϵ}}_{\text{ijk}},$, where: Y_ijk_ = observation of the i-th genotype in the j-th environment in the k-th block; μ = general constant; Gi = fixed effect of i-th genotype; B / WC_jk_ = random effect of the k-th block within WCj; WC_j_ = fixed effect of the j-th environment; GWC_ij_ = fixed effect of the interaction between the i-th genotype and the j-th WC; and ϵ_ijk_ = random error. Then, the effects of the inbred lines were estimated for each trait along with the estimates of the genotypic correlation coefficient.

## Results

### Field Study

#### Genetic Variability in Different Water Conditions and Cropped Years

The inbred lines showed significantly different agronomic characteristics in both WCs and CS, except for 100GW in WW-2018. Comparing WCs, expressive reductions (> 20%) occurred in 100GW (23.39), PE (29.31), GY (56.70), and EPV (68.27) in CS 2016 and in GY (44.58), EPV (50.61), and GNR (26.15) in CS 2018. There were significant differences between CS (except for PE), WC, and G. The CS × WC interactions for the variables GNR and ED, as well as the CS × G interactions for the variables GY, ED, and EL and the WC× G interactions for the variables 100GW, RNE, GNR, ED, and EL were not significant. The CS × WC × G interaction was significant only for PE (**Table 1**).

**Table 1:** Summary of variance analysis, general means, interactions by joint analysis for agronomic variables, SPAD index, and root architecture in popcorn inbred lines under well-watered (WW) and water stress (WS) conditions, in the 2016 and 2018 crop seasons (CS).

Traits		WS				WW				Joint analysis							
		2016		2018		2016		2018		CS	WC	G	CS^*^WC	CS^*^G	WC^*^G	CS^*^WC^*^G	
Agronomic	100-grain weight (g)	10.10	^**^	13.40	^**^	13.20	^*^	14.0	ns	^***^	^***^	^***^	^***^	^**^	ns	ns
	Popping expansion (g ml^−1^)	19.63	^**^	23.17	^**^	27.77	^*^	25.65	^**^	ns	^***^	^***^	^***^	^***^	^*^	^*^
	Grain yield (kg ha^−1^)	1089.0	^**^	1010.4	^**^	2515.2	^**^	1823.2	^**^	^***^	^***^	^***^	^***^	ns	^***^	ns
	Expanded popcorn volume (m^3^ ha^−1^)	21.88	^**^	22.49	^**^	68.96	^**^	45.54	^**^	^***^	^***^	^***^	^***^	^*^	^**^	ns
	Row number per ear	12.92	^**^	10.48	^*^	13.41	^*^	12.08	^**^	^***^	^***^	^***^	^*^	^*^	ns	ns
	Grain number per row	19.88	^**^	14.29	^**^	24.68	^**^	19.34	^**^	^***^	^***^	^***^	ns	^**^	ns	ns
	Ear diameter (mm)	27.61	^**^	25.58	^**^	29.35	^**^	28.29	^**^	^***^	^***^	^***^	ns	ns	ns	ns
	Ear length (cm)	12.40	^**^	9.51	^**^	12.31	^**^	10.77	^**^	^***^	^**^	^***^	^**^	ns	ns	ns
SPAD index	S1 (15DFA)	41.90	^**^	41.13	^ns^	40.54	^**^	39.10	ns	^*^	^**^	^***^	ns	^***^	ns	ns
	S2 (8DFA)	40.56	^**^	34.47	^*^	41.38	^**^	40.48	^**^	^***^	^***^	^***^	^***^	^***^	ns	ns
	S3 (Anthesis)	44.46	^**^	32.71	^ns^	47.30	^**^	39.60	ns	^***^	^***^	^***^	^**^	^*^	ns	ns
	S4 (7DAA)	46.98	^**^	34.45	^*^	50.29	^**^	39.93	^**^	^***^	^***^	^***^	^*^	ns	ns	ns
	S5 (17DAA)	43.90	^**^	37.53	^ns^	49.47	^**^	41.24	^**^	^***^	^***^	^***^	ns	^*^	ns	ns
	S6 (22DAA)	40.53	^**^	37.07	^**^	49.62	^**^	40.05	^**^	^***^	^***^	^***^	^***^	^**^	^*^	ns
	S7 (35DAA)	33.24	^**^	30.92	^**^	46.32	^**^	35.65	^**^	^***^	^***^	^***^	^***^	ns	ns	^**^
	S8 (42DAA)	19.62	^**^	24.65	^**^	39.91	^**^	31.44	^**^	^*^	^***^	^***^	^***^	^***^	ns	ns
Roots	Angles of brace roots (°)	52.03	^**^	62.00	^ns^	39.68	^**^	59.08	^*^	^***^	^***^	^***^	^***^	^***^	^**^	ns
	Angles of crown roots (°)	54.05	^**^	71.25	^*^	44.49	^**^	68.75	^*^	^***^	^***^	^***^	^***^	^**^	^*^	^**^
	Number of adventitious roots	10.91	^**^	12.45	^*^	10.93	^**^	12.02	ns	^**^	ns	^***^	ns	ns	^*^	^*^
	Number of brace roots	8.75	^**^	7.73	^ns^	8.43	^**^	7.73	ns	^***^	ns	^***^	ns	ns	^***^	^**^
	Number of crown roots	16.38	^**^	13.92	^ns^	15.83	^**^	14.67	ns	^***^	ns	^***^	^*^	ns	^*^	ns
	Density of adventitious roots	6.73	^**^	4.42	^**^	6.23	^**^	5.23	ns	^***^	ns	^***^	^***^	^**^	^*^	^**^
	Density of brace roots	6.39	^**^	4.35	^**^	5.33	^**^	4.93	ns	^***^	ns	^***^	^***^	^***^	^***^	^*^
	Density of crown roots	5.78	^**^	3.45	^**^	3.91	^**^	3.77	ns	^***^	^***^	^***^	^***^	^***^	^***^	ns

Levels of signification: *p < 0.05; **p < 0.01; ***p < 0.001; ns, not significant. SPAD index, expressed in days before (DBA) and after (DAA) anthesis.

Most of the inbred lines presented significantly different SPAD index values in both WCs and CS. In the evaluations conducted in 2018, the variables S1 and S3 in WS and WW, as well as S5 in WS, presented no significant differences. Comparing the WS and WW conditions, significant reductions (> 10%) occurred for S5 (11.26), S6 (18.33), S7 (28.23), and S8 (50.85) in 2016; and S2 (14.85), S3 (17.41), S4 (13.72), S7 (13.26), and S8 (21.59) in 2018. There were significant differences for CS, WC, and G when considering the SPAD variable for all evaluation dates. The CS × WC interactions for SPAD in the evaluations at S1 and S5, as well as the CS × G interactions at S4 and S7 and WC × G interactions at S1, S2, S3, S4, S5, S7, and S8 were not significant (*p* > 0.05). The CS × WC × G interaction was significant only for S7 (*p* < 0.01) (**Table 1**).

In CS 2016, the inbred lines presented significant differences in all the root characteristics and in both WCs. In 2018, the variables AC, NA, DA, DB, and DC under WS conditions, and AB and AC under WW conditions, expressed significant differences between genotypes. Comparing the WS and WW conditions, significant increases were observed in the variables AB (31.13), AC (21.48), DB (19.83), and DC (47.90) in 2016, and decreases in DA (15.61) and DB (11.82) in 2018.

Root variables presented significant differences for CS and G. The absence of significant differences for WC was observed in NA, NB, NC, and DB. The CS × WC interactions for NA and NB, as well as the CS × G interactions for NA, NB, and NC were not significant (*p* > 0.05). All root variables had significant WC × G interactions. The CS × WC × G interaction was significant for AC, NA, NB, DA, and DB (*p* < 0.01) (**Table 1**).

#### Agronomic and Root Traits Performance

**Figure 4:**
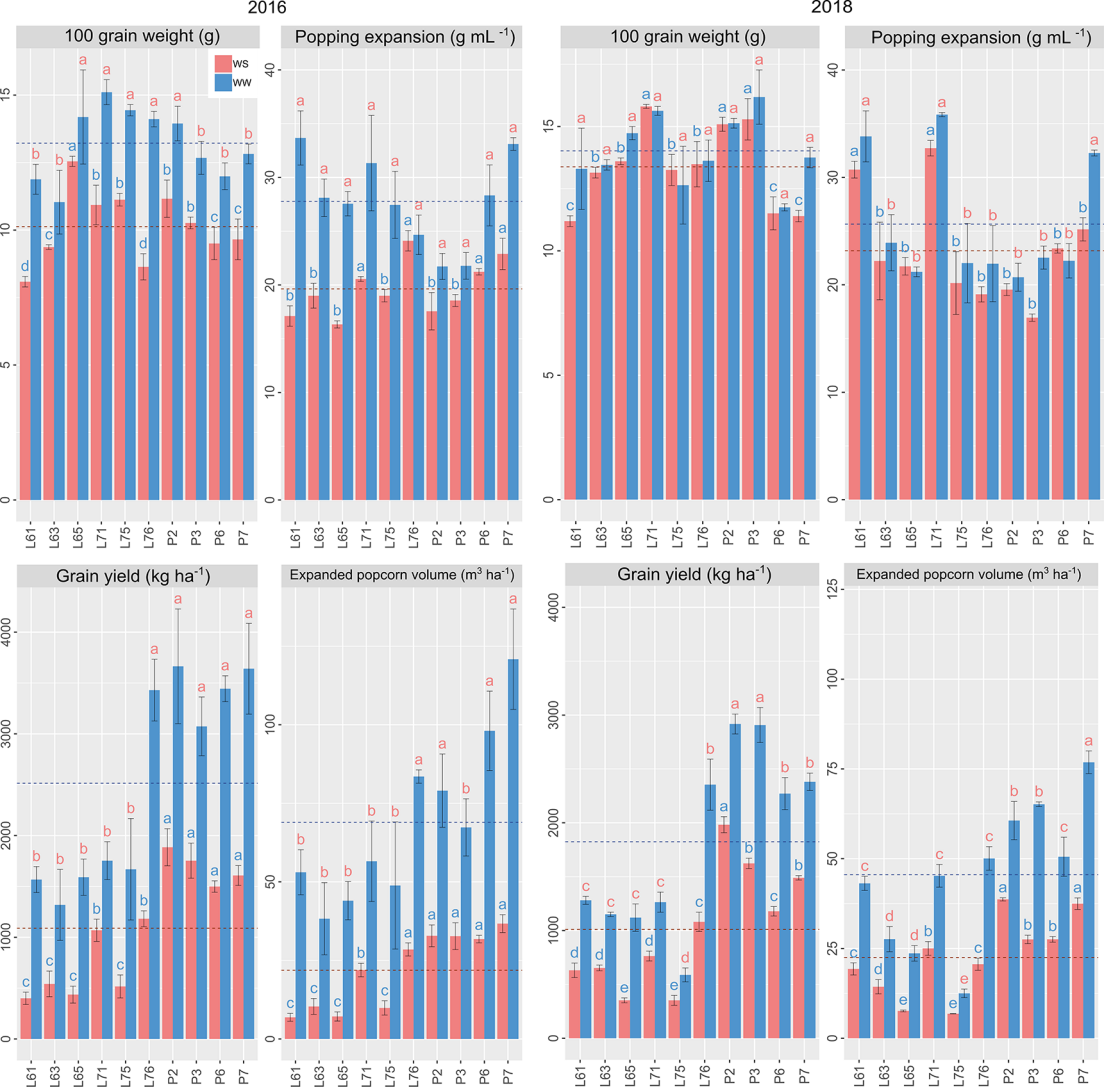
Agronomic potential of popcorn inbred lines under WS and WW conditions in 2016 and 2018. Mean genotypes, red (WS) and blue (WW), followed by the same letter do not differ using the Scott and Knott test at 5%. The bars denote the standard error.

Under WS and WW conditions for CS evaluated in relation to 100GW, L65 presented the highest mean estimates. The inbred lines L71, L76, P6, and P7 had the highest means for PE in CS 2016 and L61, L71, P6, and P7 in CS 2018. The highest GY and EPV means, at the same time for different WCs and CS, were obtained for P2, P3, P6, and P7 (**Figure 4**).

**Figure 5:**
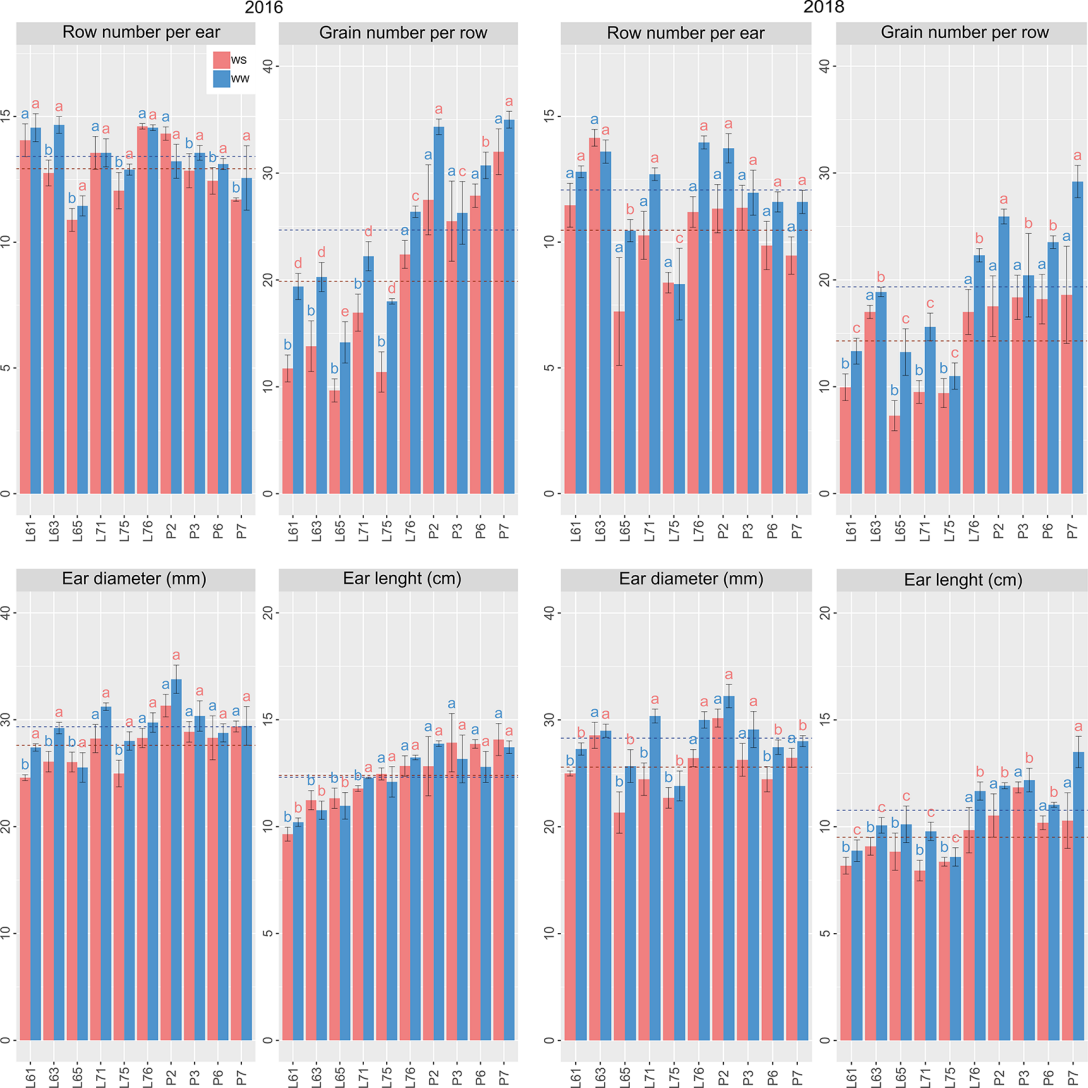
Agronomic potential of popcorn inbred lines under WS and WW conditions in 2016 and 2018. Mean genotypes, red (WS) and blue (WW), followed by the same letter do not differ using the Scott and Knott test at 5%. The bars denote the standard error.

The highest mean values for RNE, occurring simultaneously in different WCs and CS, and were expressed by L61, L71, L76, and P2; for GNR by P2 and P7; for ED by L76, P2, and P3; and for EL by P2 and P7 (**Figure 5**).

Unlike L61, L65, and L75, the P2, P6, and P7 inbred lines expressed better agronomic performance for GY and EPV in both WCs and CS. In greenhouse studies and with a greater number of physiological traits, the genotype P7 was notable as being superior and L75 as inferior.

**Figure 6:**
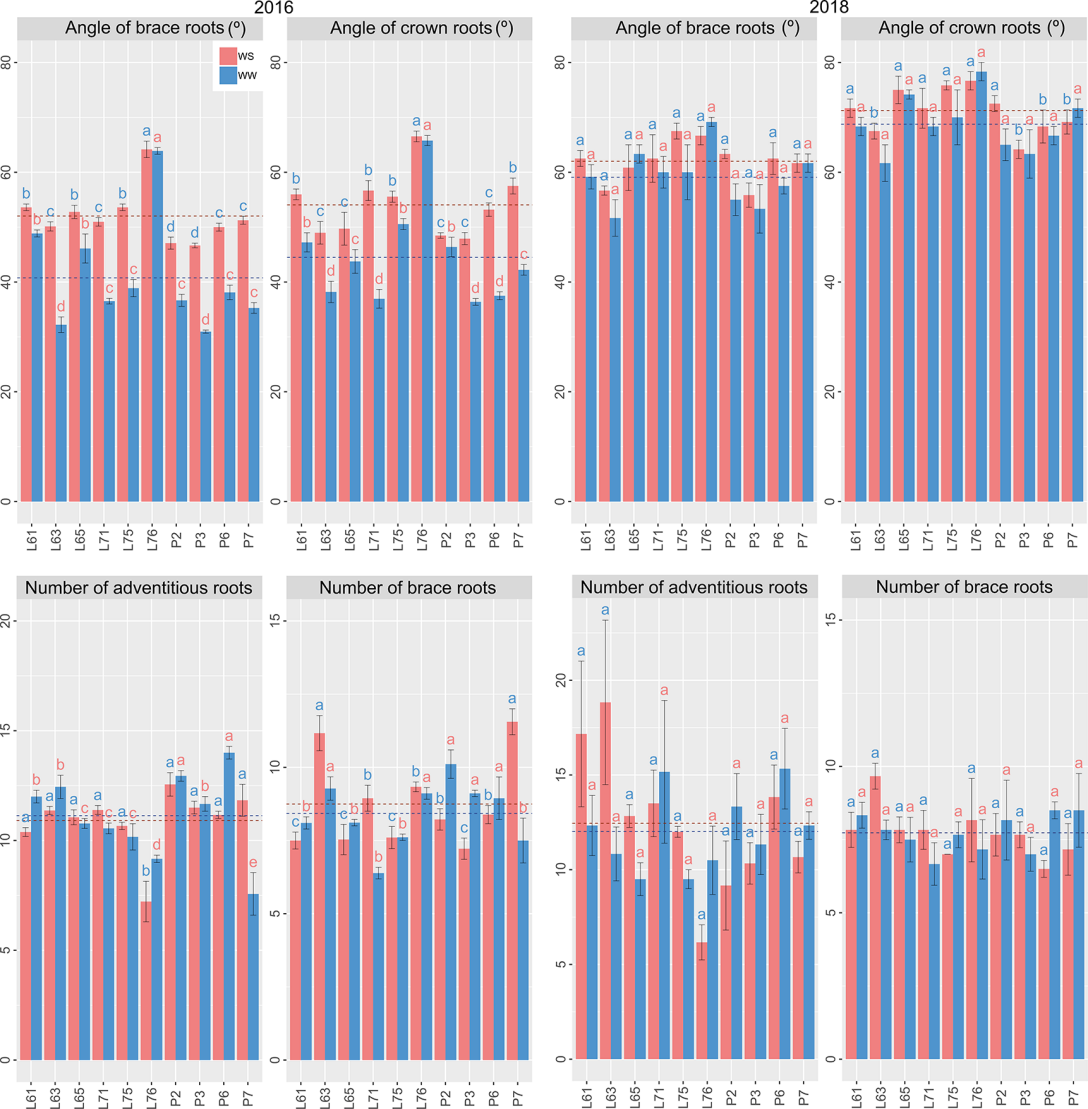
Root traits of popcorn inbred lines under WS and WW conditions in CS 2016 and 2018. Mean genotypes, red (WS) and blue (WW), followed by the same letter do not differ using the Scott and Knott test at 5%. The bars denote the standard error.

**Figure 7:**
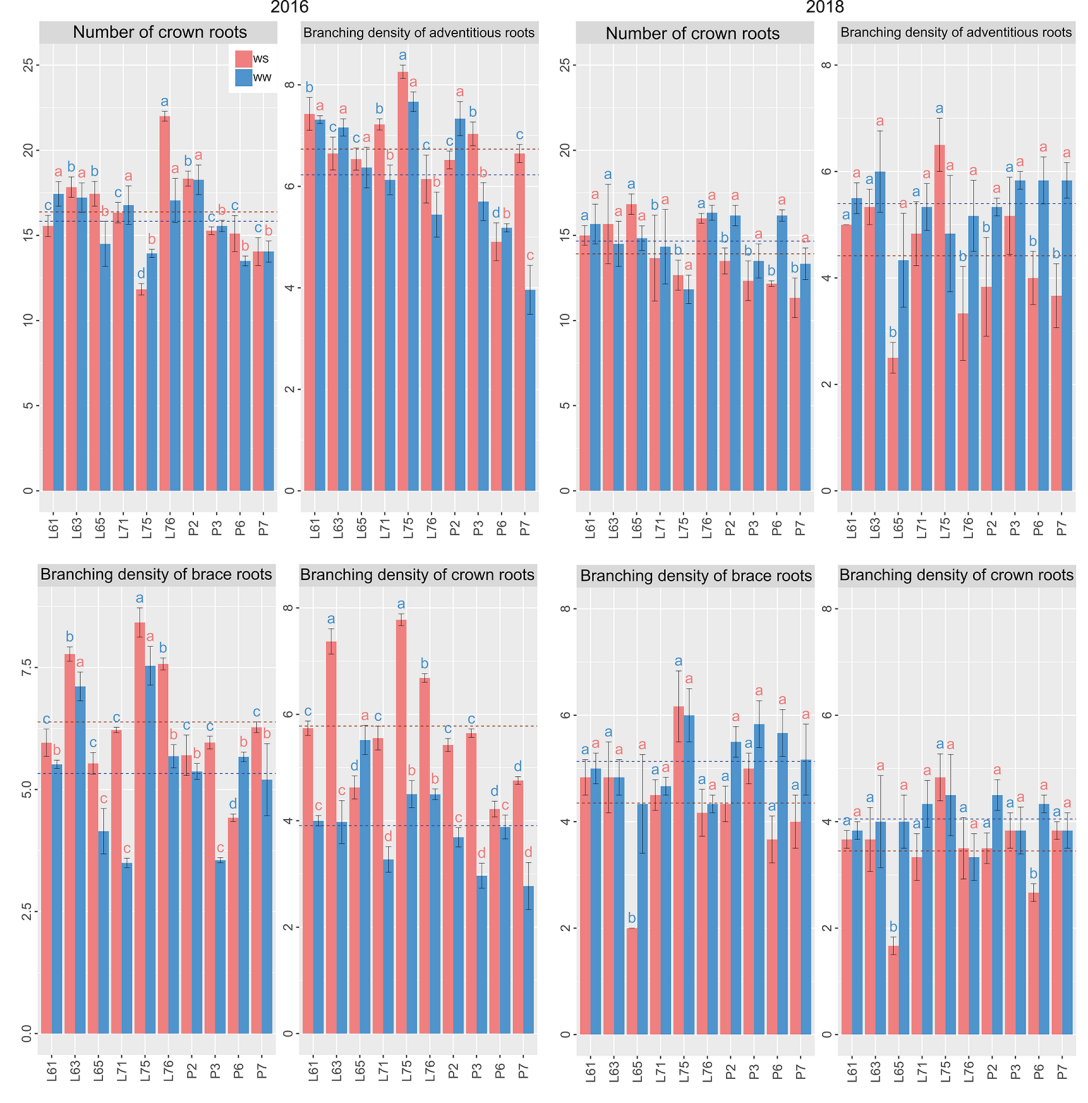
Root traits of popcorn inbred lines under WS and WW conditions in CS 2016 and 2018. Mean genotypes, red (WS) and blue (WW), followed by the same letter do not differ using the Scott and Knott test at 5%. The bars denote the standard error.

Considering the different WCs and CS together, L61, L65, and L76 showed the highest estimates for AB, whereas L61, L75, and L76 were notable with the highest means for AC (**Figure 6**). Superiority of NA was identified for P2 and P6 (**Figure 6**), whereas that of NC was identified in L76. The variables related to root density showed the supremacy of L75 (**Figure 7**).

#### SPAD Index in the Phenological Stages From Pre-Anthesis to Physiological Maturation

**Figure 8:**
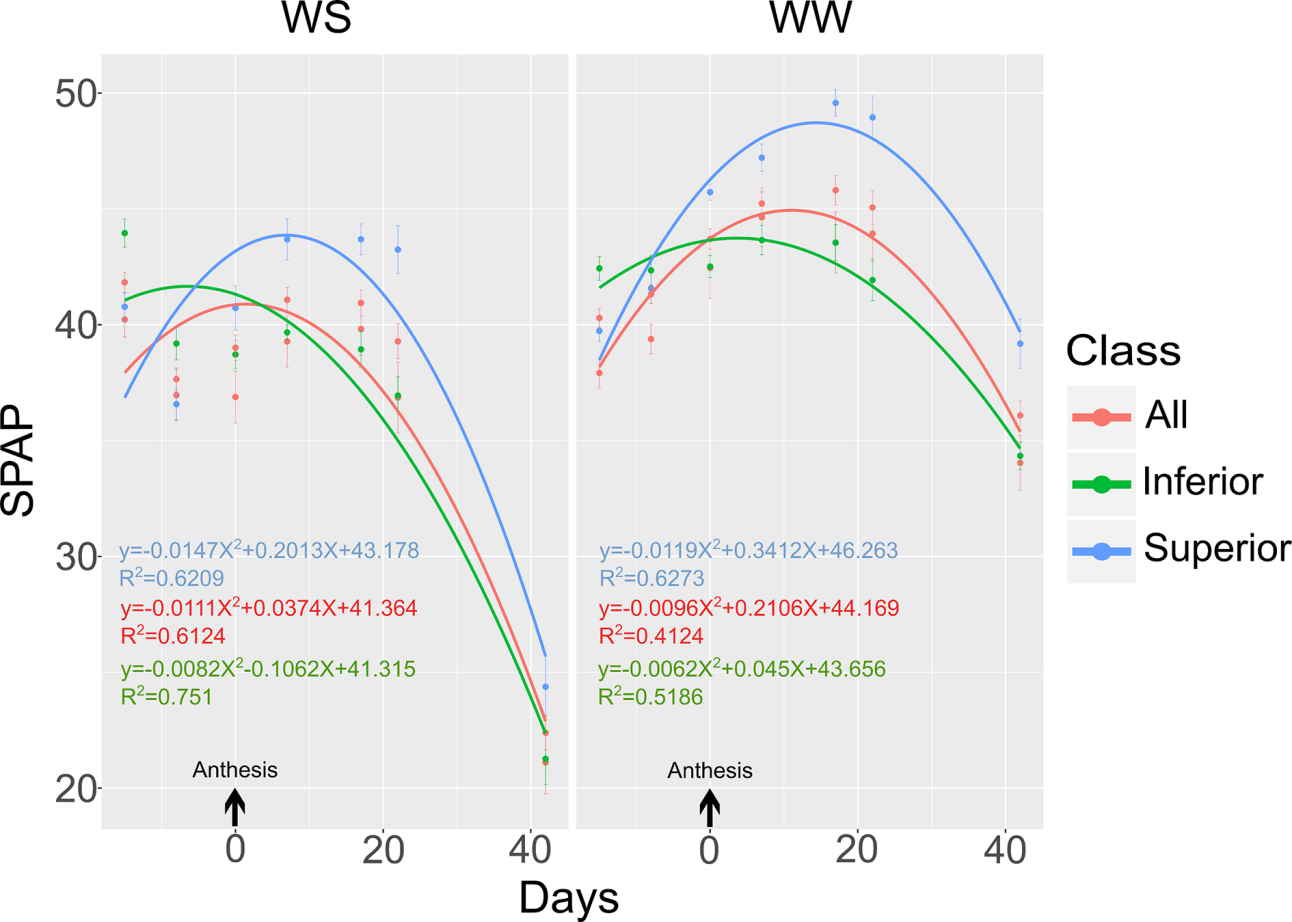
SPAD index evaluated in ten popcorn inbred lines (all) with contrasting performance groups, i.e., superior and inferior, measured at different dates from the male pre-anthesis period until physiological maturity. SPAD index: S1 (15 DBA), S2 (8 DBA), S3 (anthesis), S4 (7 DAA), S5 (17 DAA), S6 (22 DAA), S7 (35 DAA), and S8 (42 DAA).

The evaluation of S7 was removed from the analysis because the CS × WC × G interaction effect was significant (*p* < 0.01) (**Table 1**). For the other SPAD index evaluations, the means of the variable in both CS were used. The quadratic model was the best fit to the response of the SPAD index with the number of days before and after male anthesis in the different WCs (**Figure 8**). In addition, **Figure 8** shows the regressions of SPAD index values over the days before and after male anthesis as that of three groups: superior (P2, P6, and P7), inferior (L61, L65, and L75), and evaluation of all inbred lines.

Considering the inbred lines evaluated jointly (all), the value corresponding to the starting point (β_0_), date of occurrence of the male anthesis, was 41.36 in WS and 44.17 in WW. In WS, the maximum value of the SPAD index was 41.39, verified at both DAA. Whereas in WW, the maximum value observed was 45.32, verified at 11 DAA (**Figure 8**). At S8, the SPAD index values were 23.35 and 36.08 in WS and WW, respectively.

The superior genotypes had values of 41.31 in WS and of 46.26 in WW corresponding to β_0_. In WS, the maximum value of the SPAD index was 43.18, reached at seven DAA. Whereas in WW, the maximum value observed was 48.71, found at 14 DAA (**Figure 8**). At S8, the SPAD index values were 25.70 and 39.60 in WS and WW, respectively.

The inferior genotypes had values of 41.31 in WS and 43.66 in WW corresponding to β_0_. In WS, the maximum value of the SPAD index was 41.66 at six DAA. Whereas in WW, the maximum point was 43.74 at four DAA (**Figure 8**). At S8, the SPAD reading values were 22.39 and 34.61 in WS and WW, respectively.

#### Relations Between Agronomic and Root Characteristics and SPAD Readings

The variables PE, S7, AC, NA, NB, DA, and DB, which showed significant CS × WC × G interaction (**Table 1**), were eliminated from the genetic correlation analysis (*rg*).

**Figure 9:**
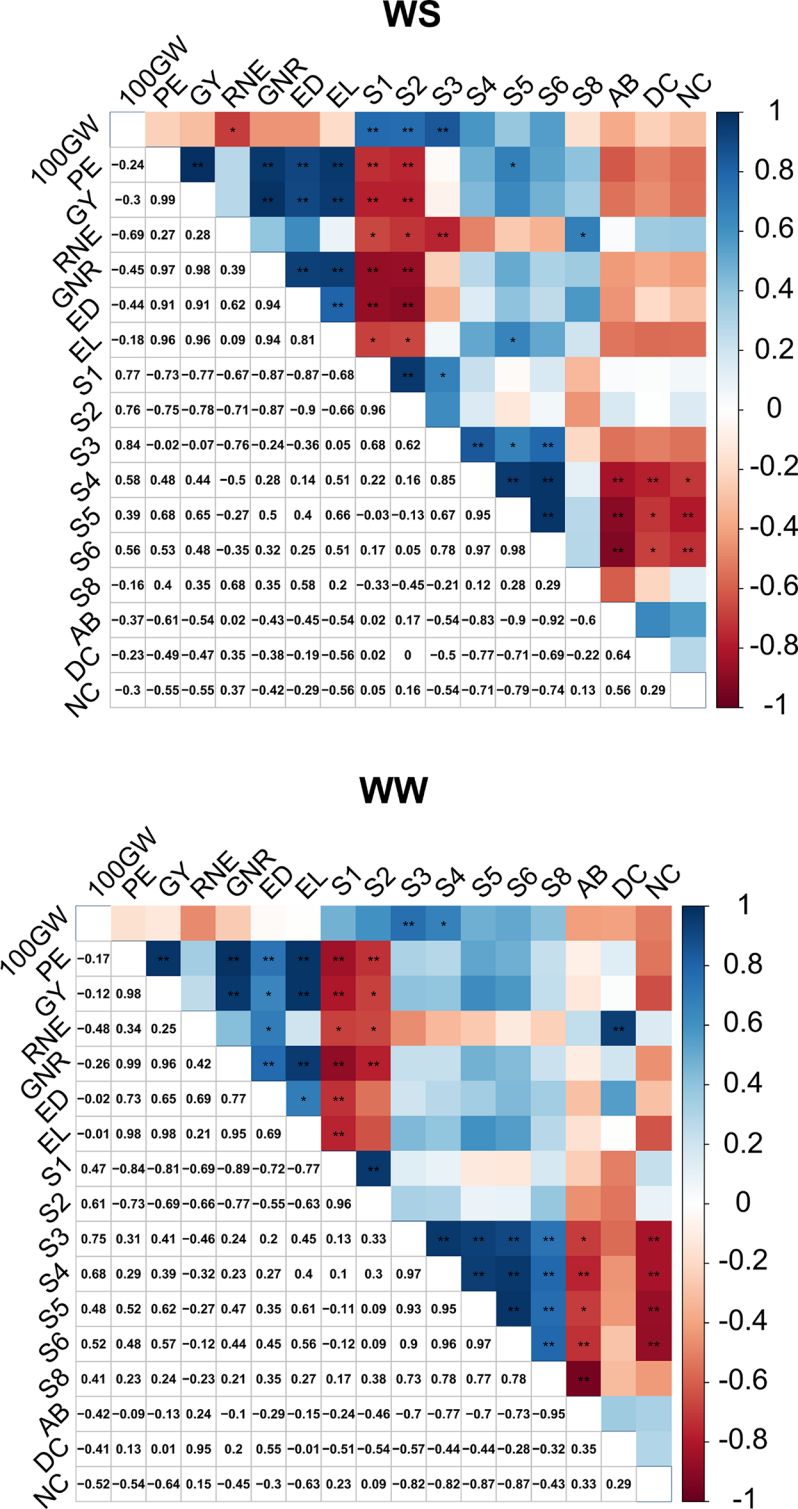
Estimates of the genotypic correlation coefficient between agronomic, root traits, and different dates of SPAD reading in popcorn inbred lines evaluated under different water conditions. 100GW: 100-grain weight, GY: grain yield, EPV: expanded popcorn volume, RNE: row number per row, GNR, grain number per row; ED, ear diameter; EL, ear length. SPAD index: S1 (15 DBA), S2 (8 DBA), S3 (anthesis), S4 (7 DAA), S5 (17 DAA), S6 (22 DAA), S8 (42 DAA). AB, angles of brace roots; NC, number of crown roots; DC, branching density of crown roots.

In the WS condition, for the main characteristics of agronomic interest, GY and EPV, there were positive estimates for the GY × EPV, GY × GNR, GY × ED, GY × EL, EPV × GNR, EVP × ED, and EPV × EL pairs. Among the SPAD index evaluations, the most expressive *rg* were in the pairs S1 × S2, S1 × S3, S3 × S4, S3 × S5, S3 × S6, S4 × S5, S4 × S6, and S5 × S6, all also positive. Although with positive estimates, no significant *rg* was observed for root variables (**Figure 9**).

Analyzing the most significant *rg* in the WS condition, the notable pairs between agronomic characteristics and the SPAD index were 100GW × S1 (positive); GY × S1, EPV × S1, RNE × S1, GNR × S1, and ED × S1 (negative); 100GW × S2 (positive); GY × S2, EPV × S2, RNE × S2, GNR × S2, ED × S2, and EL × S2 (negative); 100GW × S3 (positive); RNE × S3 (negative); GY × S5, EPV × S5, EL × S5, and RNE × S8 (positive). No significant positive *rg* were observed between agronomic and root variables, although there was a tendency for negative correlations between them. Among the different SPAD reading dates, the following negative *rg* were observed for the root variables: S4 × AB, S4 × NC, S4 × DC, S5 × AB, S5 × NC, S5 × DC, S6 × AB, S6 × NC, and S6 × DC (**Figure 9**).

In the WW condition, a positive *rg* was observed between GY × EPV, GY × GNR, GY × ED, GY × EL, EPV × GNR, EPV × ED, and EPV × EL. Among the SPAD index evaluations, there were significant and positive *rg* between S1 × S2, S3 × S4, S3 × S5, S3 × S6, S3 × S8, S4 × S5, S4 × S6, S4 × S8, S5 × S6, S5 × S8, and S6 × S8. No significant *rg* was observed for root variables, although positive *rg* occurred among them (**Figure 9**).

Analyzing the *rg* between agronomic traits and SPAD index in the WW condition, the associations GY × S1, EPV × S1, RNE × S1, GNR × S1, ED × S1, GY × S2, EPV × S2, RNE × S2, and GNR × S2 were negative, and the associations 100GW × S3 and 100GW × S4 were positive. A significant and positive *rg* was observed between the agronomic variable RNE and the root variable NC. In general, there was a tendency toward negative *rg* between the root variables AB and DC and agronomic variables. The following negative *rg* were observed between different SPAD index evaluation dates for the root variables, S3 × AB, S4 × AB, S5 × AB, S6 × AB, S8 × AB, S3 × DC, S4 × DC, S5 × DC, and S6 × DC (**Figure 9**).

### Greenhouse Study

#### Phenotyping of Contrasting Genotypes

##### Morphological Characteristics and Foliar Pigments

In the WS condition, SB was 72.40 and 49.30 g for P7 and L75, respectively; and in the WW condition, P7 and L75 presented SB of 113.67 and 79.21 g, respectively. In the different WCs, there was a significant difference between means, with a proportional variation of approximately 37.0%. Regardless of WC, P7 tended to have a lower SLA (but without achieving significance) in relation to L75. There was a mean SLA increase of approximately 21.8% when comparing WS with WW. The leaf area (LA), independently of WC, was higher in P7, with values of 0.51 m² and 0.63 m² in WS and WW, respectively. In both WCs there was a difference in the comparison between means. L75 had a greater decrease in LA, corresponding to 32.5% (**Table 2**). The joint analysis of the variables studied here presented significant differences between G and WC, without significant G × WC interaction (**Table 2**).

**Table 2:** Mean values of morphological characteristics, foliar pigments, gas exchange measurements, C isotope discrimination, and root variables in popcorn inbred lines contrasting with drought under different water conditions.

Traits	WS			WW			Joint analysis		
	P7	L75	Prob.	P7	L75	Prob.	G	WC	G*WC
Shoot biomass (g)	72.40	49.30	^***^	113.67	79.21	^*^	^**^	^**^	^ns^
Specific leaf area (cm^2^ g^−1^)	199.33	228.38	ns	163.56	187.63	ns	^*^	^**^	^ns^
Leaf area (m²)	0.51	0.37	^**^	0.63	0.54	^*^	^**^	^**^	^ns^
Stomatal density adaxial (stomata mm^−^²)	80.17	106.36	^**^	74.56	96.49	^*^	^**^	ns	^ns^
Stomatal index adaxial (%)	21.00	21.27	ns	20.88	22.26	ns	ns	ns	^ns^
Stomatal density abaxial (stomata mm^−^²)	106.73	105.26	ns	83.33	116.23	^**^	ns	ns	^ns^
Stomatal index abaxial (%)	27.47	22.85	^**^	22.39	27.71	^**^	ns	ns	^**^
Chlorophyll content (SPAD index) (arbitrary units)	27.93	30.75	ns	42.15	34.45	^*^	ns	^*^	^ns^
Flavonoid content (arbitrary units)	0.97	0.97	ns	0.96	1.05	ns	ns	ns	^ns^
Anthocyanin content (arbitrary units)	0.20	0.18	ns	0.18	0.19	ns	ns	ns	^ns^
Nitrogen balance index (arbitrary units)	29.08	31.90	ns	44.96	34.67	^**^	ns	^**^	^ns^
Net photosynthetic rate (A, µmol CO_2_ m^−2^ s^−1^)	27.50	19.80	^**^	30.13	32.33	ns>	^*^	^**^	^*^
Stomatal conductance (g_s,_ mol H_2_O m^−2^s^−1^)	0.13	0.09	^*^	0.17	0.19	ns	ns	^**^	^*^
Transpiration rate (E, mmol H_2_O m^−2^s^−1^)	3.50	2.55	^*^	4.56	4.96	ns	ns	^**^	^*^
Relative water content (%)	89.92	90.64	ns	92.44	92.04	ns	ns	ns	^ns^
WUE_Instantaneous_ (μmol CO_2_ mmol H_2_O^−1^)	7.86	7.92	ns	6.64	6.56	ns	ns	^**^	^ns^
WUE_intrinsic_ (μmol CO_2_ mol H_2_O^−1^)	218.52	213.16	ns	183.63	179.29	ns	ns	^**^	^ns^
WUE_agronomic_ (g kg^−1^ DM)	6.06	5.81	ns	5.40	6.33	ns	ns	ns	^ns^
Stable carbon isotope composition (δ^13^C) (‰)	−13.16	−13.75	^*^	−12.18	−12.37	ns	^**^	^***^	^*^
RWDa (g m^−3^)	117.53	75.78	^*^	193.21	155.22	^*^	^**^	^**^	^ns^
RWDb (g m^−3^)	44.38	27.60	^*^	65.21	55.73	ns	^*^	^**^	^ns^
RWDc (g m^−3^)	25.31	18.16	^*^	59.60	55.58	ns	ns	^**^	^ns^
RWDd (g m^−3^)	13.46	13.46	ns	51.94	33.29	ns	^*^	^**^	^*^
RWDe (g m^−3^)	21.06	17.05	ns	58.61	40.04	^*^	^*^	^**^	^ns^
Shoot/root biomass dry ratio	4.96	4.90	ns	4.06	3.53	ns	ns	^*^	^ns^

Prob.: Differences between inbred lines in each water treatment (WS, water stress; WW, well-watered) were separated by genotype effects. Levels of significance: *p < 0.05; **p < 0.01; ***p < 0.001; ns, not significant. WUE, water use efficiency; RWD, root weight density, where RWD a, b, c, d, and e refer to the depth of each soil section. G, genotypes; G*WC, genotype × water condition interaction.

Among the variables related to stomatal characteristics, significant differences were observed between adaxial stomatal density and abaxial stomatal density, in both WCs; and for abaxial stomatal density only in WW. The comparison between WCs, in general, showed increases for P7 and decreases for L75 when considering these variables. The joint analysis showed neither statistical differences between G and WS nor significant G × WC interactions (**Table 2**).

In foliar pigments, statistical differences were observed only in chlorophyll and NBI in the WW condition. Chlorophyll and NBI decreased under WW compared with WS conditions, being more pronounced for P7. However, there was also a slight increase of anthocyanin in the same lineage. The joint analysis for both WC showed statistical differences only for chlorophyll and NBI (**Table 2**).

#### Gas Exchange and Water Status

In WW, no statistical differences were observed between genotypes in A, g_s_, and E (**Table 2**). However, under WS the P7 plants showed A, g_s_, and E values of 27.50 µmol m^−2^ s^−1^, 0.13 mol m^−2^ s^−1^, and 3.50 mmol m^−2^ s^−1^, respectively, which were significantly greater than the 9.80 µmol m^−2^ s^−1^, 0.09 mol m^−2^ s^−1^, and 2.55 mmol m^−2^ s^−1^ observed in L75 plants, respectively. Furthermore, P7 presented smaller proportional decreases between WCs than L75.

RWC, WUE_instant_, WUE_intrinsic_, and WUE_agron_ did not show significant differences between water conditions in P7 and L75 plants, with the exception of WUE_agron_, which varied in a similar way in both lines (**Table 2**).

In WS, the stable isotope carbon composition (δ^13^C) of biomass was −13.16‰ for P7, and −13.75‰ for L75 (significant), which were slight lower values than the −12.18‰ and −12.37‰ observed in their respective WW plants (not significant) (**Table 2**). In the joint analysis, significant differences were observed between G and WC, as well as in the G × WC interaction (**Table 2**).

#### Root Characteristics

Regardless of water condition, P7 plants had higher RWD values in the different soil strata. Significant difference between inbred lines for RWDa in both WCs and for RWDb and RWDc in WS, as well as for RWDe in WW was observed. Considering the comparison between WCs, a mean RWD decrease was observed in the different strata, being 53.4% for P7 and 57.20% for L75.

The shoot/root ratio of dry biomass increased from 3.80 to 4.93 when comparing the WW to WS condition, without significant differences between these inbred lines.

In general, root characteristics in the joint analysis showed significant differences within G and WC. However, there was no significant G × WC interaction.

## Discussion

### Field Studies of the Effect of Soil Water Limitation on Evaluated Characteristics and Implications for Plant Improvement

Soil water limitation most affected GY and EPV yield components. Water limitation implemented during pre-anthesis and grain filling reduced the yield covariates to a lesser extent. Under dry conditions, the reduction in the number of grains per ground area has been indicated as being responsible for GY reduction in common maize (Cairns et al., 2012). Pollen viability and zygote formation are drought-sensitive processes (Zinselmeier et al., 1995), leading to a reduction in the number of grains produced under water limiting conditions. This effect may have occurred in 2018, where WS was evaluated earlier (before anthesis), affecting the GNR. However, in 2016, where the water limitation occurred later, the main effect was noticed in 100GW (but not for the variables related to grain quantity), which also affected GY. Water stress may shorten the period of grain filling, decrease photosynthetic activity, accelerate ABA-mediated leaf senescence and reduce the activity of enzymes involved in sucrose metabolism and starch synthesis, which altogether can contribute to a lower grain weight (Yang and Zhang, 2006; Sehgal et al., 2018; Yang et al., 2019).

The variables of economic interest, i.e., PE, GY, and EPV, had a significant WC × G interaction, which pointed to different responses among the evaluated genotypes in the different implemented WCs. Interactions of this nature interfere with selection gains and cultivar recommendation for specific environments (Hallauer et al., 2010). In these cases, indirect selection variables that are determinants of the expression of the main characteristics and that do not present significant WC × G but are associated in a significant and positive way can be identified (Kamphorst et al., 2019). The absence of significant WC × G interaction in relation to 100GW, RNE, GNR, ED, and EL allowed us to infer that selection in any WC can be effective in obtaining simultaneous genetic gains for these characteristics.

The SPAD index can be used for the indirect selection of the most productive plants in the WS condition, as there was no significant WC × G or CS × WC × G interaction, whereas a positive association with GY and EPV was found (**Table 1**). The use of the SPAD index may allow for the reliable selection of productive genotypes both under adequate and reduced water availability environments, with “stay-green” cultivars being the best option for the latter (Joshi et al., 2006; Costa et al., 2008).

Root systems had phenotypic plasticity in different WCs, which implies significant WC × G and CS × WC × G interactions. Therefore, in addition to the difficulty in measuring root traits, the occurrence of interactions is an obstacle to improving the use of root characteristics in plant breeding (Lynch, 2015), which is reinforced by their low heritability estimates (Trachsel et al., 2011). The phenotypic plasticity of root systems was observed when there was an increase in the angles and density of the supporting roots and the crown in 2016 only. In fact, root angle increases are considered an adaptive response to water stress (Trachsel et al., 2011; Kamphorst et al., 2019).

### Dynamics of Foliar Senescence Between Contrasting Genotypes

To understand the physiological traits inherent to the most productive lines under water limiting conditions, the genotypes were grouped using on the best results for GY and EPV. Soil water depletion reduced the SPAD index regardless of genotypic groups. Foliar senescence induction is a plant response to water deficit conditions (Ghannoum, 2008; Araus et al., 2018), being coordinated by the reproductive stage of plants, but also strongly influenced by the environment (Combe and Escobar-Gutiérrez, 2009). A portable chlorophyll meter (SPAD) can be an interesting tool for plant stress diagnosis (Romano et al., 2011; Araus et al., 2012; Zia et al., 2013; De Castro et al., 2014). In fact, spectrometric methods can be used to efficiently evaluate different WCs and assess the agronomic performance of cereals (Yousfi et al., 2019).

Regardless of the phenotypic class, the maximum SPAD index values were attained later (11 DAA) in WW than in WS (2 DAA) condition. Furthermore, the genotypes with greater productivity were more stay-green (higher SPAD index values), as shown by the higher SPAD values recorded during grain filling. In contrast, genotypes with lower productivity reached higher SPAD index values earlier, irrespective of water conditions, thus reflecting an association between higher SPAD index values and higher yield values. These finding were in line with reports that higher SPAD index values, together with a senescence delay, are likely to contribute to the maintenance of C-assimilation and the mobilization of photoassimilates to the reproductive organs, therefore, increasing agricultural productivity (Gregersen et al., 2008).

### Implications of Genetic Associations Between Agronomic and Root Characteristics and the Different SPAD Measurement Dates

The GNR, ED, and EL components are important selection characteristics for yield and EPV increments, especially in popcorn plants under adequate water availability (Cabral et al., 2016; de Lima et al., 2016; do Amaral Júnior et al., 2016) and during droughts (Kamphorst et al., 2019). A disadvantage of using these characteristics is the need to wait until the end of the crop cycle to obtain results. An ideal scenario is the possibility of predicting higher yields through the use of characteristics and methods that can be evaluated early using non-destructive procedures, such as remote sensing (Araus et al., 2018).

The choice of an appropriate phenological stage for SPAD index measurements is crucial, since phenotyping methodologies should be used in critical stages associated with grain production (Cairns et al., 2012). In this study, the range of 17 to 22 DAA was adequate for these measurements, allowing us to obtain highly positive correlations to yield estimates (and its components). Under WS, maize genotypes with delayed leaf senescence are often more productive, showing selection advantages for water limiting conditions (Cairns et al., 2012; Adebayo et al., 2014). It is important to mention that among the popcorn varieties evaluated, the inbred lines that had high SPAD index values before anthesis were not the most productive. Regardless of water availability level, the high SPAD index values found at 15 and 8 DBA were negatively correlated with GY and EPV. As stated, late leaf senescence produces photoassimilates and remobilizes nutrients at the appropriate time of grain filling, thus sustaining higher yields (Jordan et al., 2012), but late remobilization can also decrease grain filling (Sehgal et al., 2018).

A root system adapted to specific abiotic soil stress conditions represents an agronomic advantage with positive impact on yield (Trachsel et al., 2011). Notably, no significant correlations were found between GY and its covariables for the evaluated root characteristics. An explanation for this can be the phenotypic plasticity of the root response, which resulted in significant WC × G interaction. However, there is a tendency for negative correlations between agronomic and root characteristics, especially with NC and DC. In fact, it was previously reported that a reduced NC led to a deeper root system and resulted in better soil water acquisition, which in turn increased carbon gain, growth and plant productivity (Gao and Lynch, 2016). An optimal number of crown roots can also interact with other characteristics to which they are associated, and this will increase deeper soil exploration (increases tolerance to water deficit), such as the greater root angle in relation to the soil surface and reduced lateral branching (Trachsel et al., 2013; Zhan and Lynch, 2015; Zhan et al., 2015).

The highest SPAD values at 7, 17, and 22 DAA were associated with the lowest root values for the variables AB and DC, regardless of water availability. As for the NC variable in WS, there was a negative association with the same SPAD measurement dates (7, 17, and 22 DAA). In fact, the lowest NC value, as well as the decreased branching density of the lateral root, may have improved the water absorption capacity in the soil, which was indirectly reflected in a better leaf status and therefore higher SPAD values. Notably, the most productive genotypes under lower water availability are expected to have steeper angles to the soil horizon (Gao and Lynch, 2016), which was not observed in our study.

### Phenotyping of Contrasting Genotypes Under Greenhouse Conditions

When comparing the two water availability levels, the superior genotype (P7) showed higher SB and LA values, and a smaller proportional LA decrease under WS when compared to WW. Higher biomass accumulation before flowering and increased leaf size may result in a larger photosynthesizing area, which would increase C-assimilation (Tollenaar et al., 2004). SLA showed significant and positive correlations with water use efficiency, expressed by the ratio of plant dry matter to water used (Zhang et al., 2015). These authors showed that there was an increase in SLA values in maize plants at the seedling stage, while in the booting and tasseling phases, SLA decreased, coinciding with the translocation of photoassimilates to the stem and reproductive organs. In our study, the plants under both water regimes showed a significant and positive correlation of SLA with the agronomic efficiency for water use (**Supplementary Figure 1**). However, this did not contribute to the differentiation of the tested genotypes, but only the water conditions.

Water regime did not affect stomatal density or index of the abaxial and adaxial leaf sides. However, following WS, P7 had lower stomatal density than L75 on the adaxial face. As a response to water stress, the stomatal density and the stomatal index decreased in L75, considered the most sensitive line. Stomatal density and index in combination with the stomatal opening can determine the maximum diffusive conductivity of the leaf to CO_2_ (Brodribb et al., 2013; Zhao et al., 2015). In maize plants, decreased soil water availability was observed to significantly increase stomatal density, together with a reduction of stomatal size and opening (Zhao et al., 2015). However, our results indicate higher net photosynthetic rate (A) and transpiration rates in leaves exhibiting a lower density and stomata index, which may be associated with the compensatory effect of stomata size (i.e., larger stomata area, which was not measured in this study). It is important to note that g_s_ is considered a key trait for the detection of a WS effect (Hetherington and Woodward, 2003). In fact, Zhao et al. (2015) also reported that photosynthetic rate and transpiration were negatively correlated with stomatal density in maize plants. However, under WS, P7 plants showed higher A values and transpiration than L75, associated to a higher g_s_ value (**Table 2**). Gas exchange measurements differentiate inbred lines in WS, where P7 presented higher A, g_s_, and E values. Also, limited water in the soil caused a reduction of only 9% in A for P7, reflecting a minor impact in the functioning of the photosynthetic apparatus. The maintenance of higher A values for P7 than L75 in WS conditions, might have been supported by maintaining chlorophyll pigment concentration (Busch et al., 2009). Thus, the superiority of P7 is explained by the higher SB and LA accumulation.

P7 plants maintained the highest g_s_ value, even with lower adaxial stomatal density, showing a greater ability to obtain water from the soil to maintain water status under WS, as suggested by the higher RWD values in layers a, b, and c. Roots play an important role in plant adaptation to dry environments, and genotypes that have a more developed root system can provide higher GY (Ali et al., 2016). Lambers et al. (2002) reported that the metabolic cost of the root system for soil exploration under dry conditions is high, which can exceed the plant’s daily photosynthetic rate by 50%. Thus, higher A values in P7 plants might have supported the costs for this larger root system.

Under soil water limitation conditions, amongst other strategies, tolerant genotypes can maintain a higher RWC than more sensitive ones, associated to larger and more efficient root systems (increasing water removal from the soil) or by osmotic adjustment (Blum, 2011). The higher RWC results in improved cellular homeostasis and greater stomatal opening, which collectively allows the photosynthetic metabolism to be maintained to some extent (Blum, 2011). However, the ability to keep the stomata open can increase water loss *via* transpiration, so that RWC is reduced. In fact, there was a weak negative correlation between g_s_, E, and RWC in the WS condition (**Supplementary Figure 1**). Even so, A and g_s_ had positive correlation with several root traits under WS, but not under WW conditions. Interestingly, A and g_s_ had significant and positive correlation in WS but did not show the same result in WW. These results indicate an adjustment associated mainly with stomatal density in the WS condition, as observed by the strong negative correlation between g_s_ and SDA (**Supplementary Figure 1**). Both WUE_instant_ and WUE_intrinsic_ increased equally among the inbred lines under WS. This finding indicates that the lower stomatal conductance and transpiration rates influenced the use of H_2_O in the same manner. However, WUE_instant_ and WUE_intrinsic_ did not have a positive or significant correlation with A and g_s_ in WS but presented a negative correlation in the WW condition.

WUE_intrinsic_ and WUE_instant_, together with WUE_agron_ and RWC, did not help to differentiate the evaluated inbred lines. In fact, WUE_instant_ and WUE_intrinsic_ are leaf scale characteristics mainly associated with transpiration. Therefore, an increase in these variables can be associated with reduced g_s_, which in turn can reduce photosynthesis, growth, and productivity (Blum, 2009). Therefore, WUE_agron_ has been suggested to be a variable more associated with the effective use of water (Blum, 2009). However, this variable was also not efficient in differentiating the inbred lines studied here.

In addition, as a response to WS, L75 presented lower values, i.e., more negative of δ^13^C (carbon isotope composition) than P7, indicating that L75 stomata were more frequently closed throughout the crop development (Farquhar et al., 1989; Cabrera-Bosquet et al., 2009; Araus et al., 2010; Cabrera-Bosquet et al., 2017), showing that L75 was more susceptible to water stress than P7.

### New Players

This study was conducted in two parts. Firstly, field experiments (in 2016 and 2018) were designed to understand which secondary characteristics were most important to explain the higher yield and EPV values (main characteristics). As a result, we identified GNR, ED, and EL, as adequate traits for plant selection. With the same objective, we sought to identify important root characteristics. Among these, at the 25 cm depth evaluated, it was not possible to diagnose root characteristics that could be indicative of satisfactory agronomic performance. The high root plasticity response to different WCs may have been responsible for this finding. There was an association between the highest SPAD index values and the highest yield values, favoring the use of this characteristic for indirect selection. Second, complementing the field experiments, two contrasting inbred lines were studied under greenhouse conditions. Under WS at the end of the experiment, P7 maintained higher values of leaf net photosynthetic rates, stomatal conductance, and transpiration than L75. Thus, single-leaf gas exchange measurements shortly before the harvest proved to be adequate indicators for the evaluation the agronomic performance of popcorn under soil water limitation conditions. However, gas exchange measurements are not amenable for high throughput phenotyping when a large number of genotypes are evaluated. In that case, a trait which can be used to evaluate stomatal conductance throught the crop cycle, such as the stable carbon isotope composition on dry leaf matter, was shown to indicate that L75 maintained the stomata closed for longer periods (i.e. exhibited more negative value δ^13^C) under water stress than P7.

## Data Availability Statement

The raw data supporting the conclusions of this article will be made available by the authors, without undue reservation.

## Author Contributions

Conceptualization: SHK. Methodology and investigation: SHK, VJL, PHADS, WPR, JMSV, GMBG, KFMS, JTL, MV, FM-P, and OV-D. Software: VJL and PHADS. Resources: SHK, ATAJ, and JCR. Writing—original draft preparation: SHK, ATAJ, WPR, JLAO, JCR, and EC. Supervision: ATAJ, JLAO, and EC. Critical review of final manuscript version: JLAO and JCR.

## Funding

This study was financed in part by the Coordenação de Aperfeiçoamento de Pessoal de Nível Superior – Brasil (CAPES) – Finance Code 001. Funding was also provided by FAPERJ, with the project E26/201.813/2017 to SK and E26/202.761/2017 to AA. Funding to JR by Fundação para a Ciência e a Tecnologia (FCT), Portugal, through the research units UID/UIDB/00239/2020 (CEF), and UIDP/04035/2020 (GeoBioTec) is also greatly acknowledged. JA acknowledges the support of Catalan Institution for Research and Advanced Studies (ICREA, Generalitat de Catalunya, Spain), through the ICREA Academia Program.

## Conflict of Interest

The authors declare that the research was conducted in the absence of any commercial or financial relationships that could be construed as a potential conflict of interest.

## Supplementary Material

Supplementary Table 1 Precipitation records and irrigation (mm) applied to popcorn inbred lines for experiments conducted during 2016 and 2018 in WS and WW conditions in relation to days after sowing (DAS).

Supplementary Figure 1 Correlation coefficients between morphological traits (SB, SLA, LA, SDAd, SIAd, SDAb, and SIAb), foliar pigments (Chl, Flav, Anth, NBI), gas exchange measurements (A, g_s_, and E), water status traits (RWC, WUE_instant_, WUE_intrin_, and WUE_agron_), C isotope composition (δ^13^C) and root variables (RWDa-b-c-d-e and Shot root).

## Abbreviations

100GW, 100-grain weight; A, net photosynthetic rate; AB, angles of brace roots; AC, angles of crown roots; CS, crop seasons; DA, branching density of adventitious roots; DAA, days after anthesis; DAS, days after sowing; DB, branching density of brace roots; DC, branching density of crown roots; E, transpiration rate; ED, ear diameter; EL, ear length; EPV, expanded popcorn volume; FC, field capacity; G, genotype; GNR, grain number per row; g_s_, stomatal conductance; GY, grain yield; LA, leaf area; NA, number of adventitious roots; NB, number of brace roots; NC, number of crown roots; PE, popping expansion; rg, genotypic correlation coefficient; RNE, row number per ear; RWD, root weight density; SB, shoot dry biomass; SLA, specific leaf area; SPAD, soil plant analyzer development; WC, water condition; WS, water-stressed; WUE_agron_, agronomic water use efficiency; WUE_instant_, instantaneous water use efficiency; WUE_intrinsic_, intrinsic water use efficiency; WW, well-watered.
