# Supplementary material for: Comparison of Selection Traits for Effective Popcorn (Zea mays L. var. Everta) Breeding Under Water Limiting Conditions
Source: Front Plant Sci. 2020 Aug 27;11:1289. doi: 10.3389/fpls.2020.01289 (PMC7481401; doi:10.3389/fpls.2020.01289)
Supplement: Supplementary Table 1 — Precipitation records and irrigation (mm) applied to popcorn inbred lines for experiments conducted during 2016 and 2018 in WS and WW conditions in relation to days after sowing (DAS). [file Table_1.docx]

| DAS | 2016 | | |  | 2018 | | |
| --- | --- | --- | --- | --- | --- | --- | --- |
|  | Precipitation | WS | WW |  | Precipitation | WS | WW |
| 7 | - | 13 | 12 |  | 17 | 6 | 6 |
| 14 | 26 | 8 | 8 |  | 6 | 10 | 11 |
| 21 | 7 | 9 | 11 |  | - | 10 | 10 |
| 28 | 7 | 10 | 6 |  | 11 | 10 | 11 |
| 35 | - | 6 | 6 |  | 5 | 8 | 8 |
| 42 | 1 | 13 | 13 |  | 2 | 12 | 12 |
| 49 | 18 | 1 | 3 |  | - | 12 | 13 |
| 56 | 20 | - | 4 |  | - | - | 11 |
| 63 | - | - | 10 |  | - | - | 19 |
| 70 | 15 | - | 8 |  | - | - | 19 |
| 77 | 4 | - | 7 |  | 31 | - | 1 |
| 84 | - | - | 14 |  | - | - | 17 |
| 91 | - | - | 12 |  | - | - | 14 |
| 98 | - | - | 13 |  | 65 | - | 2 |
| 105 | 24 | - | 10 |  | - | - | 14 |
| 112 | 4 | - | - |  | 9 | - | 10 |
| 119 | 7 | - | - |  | 2 | - | 10 |
|  | 133 | 60 | 138 |  | 148 | 69 | 187 |
