# Supplementary figures and images for: Comparison of Selection Traits for Effective Popcorn (Zea mays L. var. Everta) Breeding Under Water Limiting Conditions
Source: Front Plant Sci. 2020 Aug 27;11:1289. doi: 10.3389/fpls.2020.01289 (PMC7481401; doi:10.3389/fpls.2020.01289)

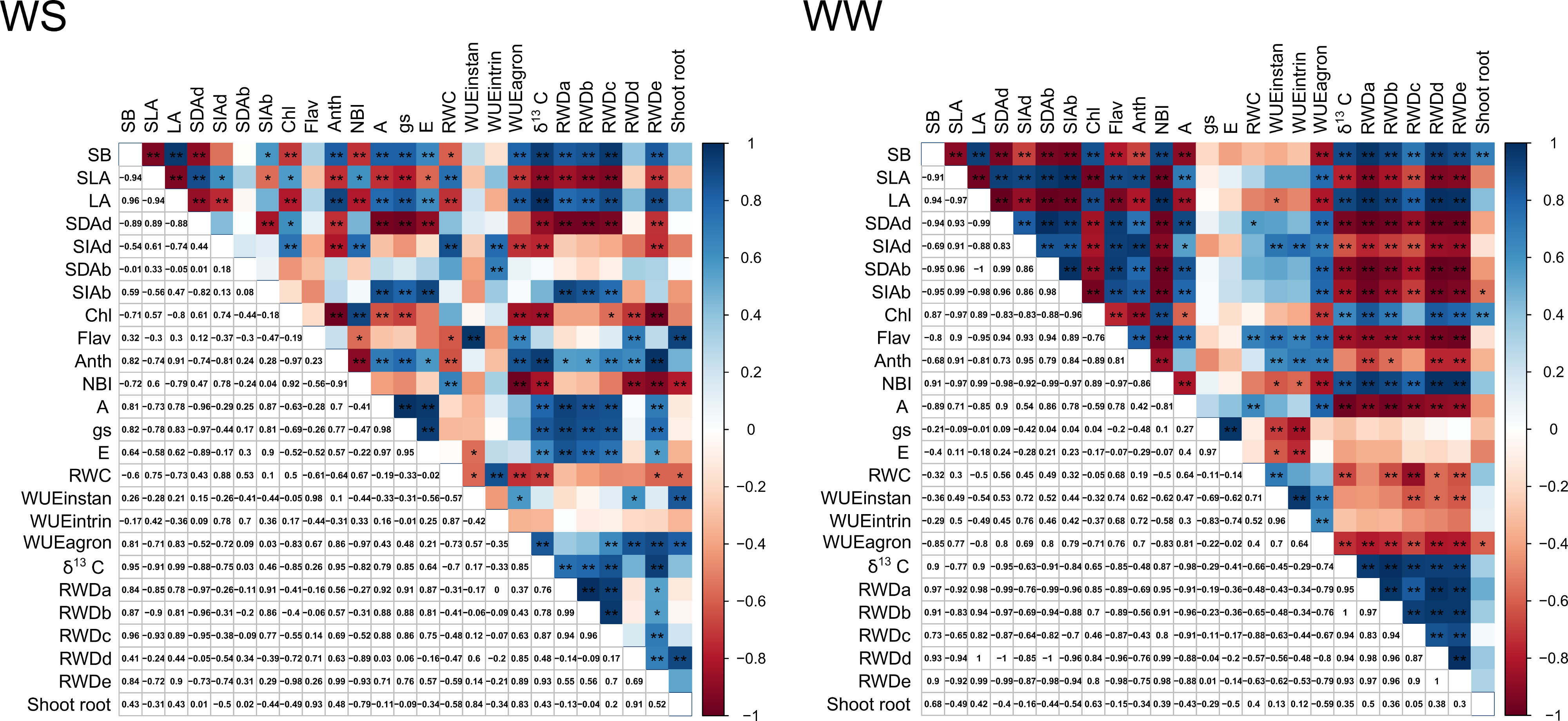

Supplement: Supplementary Figure 1 — Correlation coefficients between morphological traits (SB, SLA, LA, SDAd, SIAd, SDAb, and SIAb), foliar pigments (Chl, Flav, Anth, NBI), gas exchange measurements (A, gs, and E), water status traits (RWC, WUEinstant, WUEintrin, and WUEagron), C isotope composition (δ13C) and root variables (RWDa-b-c-d-e and Shot root). [file Image_1.tiff]
